# Leaching Rate of Polychlorinated Biphenyls (PCBs) from Marine Paint Chips

**DOI:** 10.1007/s00244-021-00868-6

**Published:** 2021-07-01

**Authors:** Allen D. Uhler, Jeffery H. Hardenstine, Deborah A. Edwards, Guilherme R. Lotufo

**Affiliations:** 1 NewFields Environmental Forensics Practice, LLC, 300 Ledgewood Place, Rockland, MA 02730 USA; 2 ExxonMobil Environmental and Property Solutions Company, Spring, TX 77389 USA; 3 United States Army Engineer Research and Development Center, Vicksburg, MS 39180 USA

## Abstract

Polychlorinated biphenyls (PCBs) were added to certain marine vessel bottom paints as a plasticizer to improve the adhesion and durability of the paint. The most common PCB formulation used to amend such paints was Aroclor 1254. Fugitive Aroclor-containing paint chips generated from vessel maintenance and repair operations represent a potential source of PCB contamination to sediments. Limited published studies indicate that Aroclor-containing paint is largely inert and exhibits low PCB leaching into water; however, the rate and degree of leaching of PCBs from paint chips have not been directly studied. This laboratory-based study evaluated the rate and extent of leaching of PCBs from paint chips into freshwater. The results of this investigation demonstrate that the rate of PCB dissolution from paint chips decreased rapidly and exponentially over time. Based on this study, it is estimated that the rate of leaching of PCBs from paint chips would cease after approximately 3 years of exposure to water. When all leachable PCBs were exhausted, it is estimated that less than 1% of the mass of PCBs in the paint chips was amenable to dissolution. The results of this experiment suggest that Aroclor-containing paint chips found in sediments are likely short-term sources of dissolved-phase PCB to pore or surface waters and that the majority of the PCBs in paint chips remain in the paint matrix and unavailable for partitioning into water.

**Supplementary Information:**

The online version contains supplementary material available at 10.1007/s00244-021-00868-6.

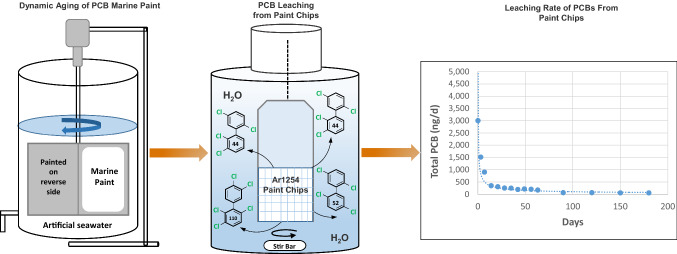

PCBs are a class of synthetic chlorinated organic compounds that, due to their chemical and physical stability, were used in a variety of commercial and industrial applications around the world beginning in 1929 and, in the USA, ending in 1977 (EPA 2013). PCBs occur as mixtures of chlorinated biphenyl molecules containing from one to up to ten chlorines per molecule. There are 209 possible congeners of chlorinated biphenyl congeners. PCBs were manufactured and marketed in the USA and other countries and sold as mixtures with a variety of trade names, including Aroclor, Pyranol, Pyroclor (USA), Phenochlor, Pyralene (France), Clopehn, Elaol (Germany), Kanechlor, Santotherm (Japan), Fenchlor, Apirolio (Italy), and Sovol (USSR) (World Health Organization 2003).

The work described herein focuses on the PCB formulation known as Aroclor 1254, manufactured by the USA-based Monsanto Industrial Chemical Company (Monsanto). Monsanto manufactured a range of PCB products under the trade name “Aroclor,” among the most common being Aroclor 1242, Aroclor 1248, Aroclor 1254, Aroclor 1260, Aroclor 1262, and Aroclor 1268. Each Aroclor product contained a subset of PCB congeners and was formulated to contain a specific relative amount of chlorine by weight, resulting in products with different physical characteristics that were found useful for a range of different commercial and/or industrial applications (Johnson et al. 2006). Aroclor 1254 which was the most common PCB plasticizer added to paints during the 1950s, 1960s, and early 1970s (Scott and Snyder 2015).

Aroclors were components of certain marine vessel paints from the 1940s until approximately mid-1971, when Monsanto, the sole U.S. PCB producer, voluntarily terminated sales of Aroclors for all but closed electrical systems (U.S. EPA 1976). Concern for the potential ecological and human health effects of PCBs led to a Federal ban on the manufacture of PCBs in the USA in 1977.

Historically, Aroclors were widely used as plasticizers in construction materials, including as additives to paint and surface coatings. Aroclors were added to ship paints to give the paints better adhesive properties and to provide added protection from corrosion, chemicals, and flames (Jensen et al. 1972; Martin and Richards 2010). Chemical-resistant chlorinated rubber paints were formulated with various Aroclors, typically at 10–12% (dry weight) of the total composition (Scott and Snyder 2015). Construction materials containing Aroclors were widely used in industrial and institutional settings (Scott and Snyder 2015; Jartun et al. 2009), but also in construction and maintenance of military and civilian vessels. Aroclors were added to marine paint used to protect boats starting in the 1940s until approximately early 1971. Chlorinated paraffins replaced Aroclor-containing paints, as the post-production stock of Aroclor-containing paints was consumed (Martin and Richards 2010).

Many vessel maintenance facilities have been sites of ship refurbishing and application of paints and coating materials on decks, cabins, and hulls of vessels to provide protection against erosion, corrosion, and biofouling. Historic sand blasting practices, commonly used to remove old paint and fouling organisms from hulls, vessel spray painting, and flaking of old paint are potential sources of paint, sand blast material, and related debris to the surrounding environment (Turner 2010).

Contamination of sediments proximal to harbors has been attributed to the release of various chemical constituents found in marine paints (Thomas et al. 2002; Singh and Turner 2009; Eklund and Eklund 2014; Wu et al. 2016). The greatest load of contaminants discharged from vessel maintenance activities has been associated with paint particles that deposit to the surrounding sediment beds (Turner 2010; Thomas et al. 2002; Thomas, 2003; Harris et al. 1991). Release of spent paint residues from ship repair yards and direct release from the hull during port calls were responsible for introduction of various contaminants, including PCBs, to sediments outside of ship repair yards and around quay structures in Norway (Johnsen and Engoy 2000).

Regardless of the evidence for the likely release of Aroclor-containing paint particles into aquatic environments, there are virtually no published studies regarding the fate and behavior or potential for benthic bioaccumulation for paint-associated PCBs. The lack of information hinders the ability to assess the potential environmental effects of Aroclor-containing paint particles in areas where their release to sediments has occurred.

The work described herein is intended to contribute relevant information regarding the leaching behavior and bioavailability of PCBs from Aroclor-containing paint chips. In this study, the dissolution of PCBs from paint chips was studied in a controlled laboratory setting; all 209 PCB congeners were monitored in the leachate over a 180-day leaching period.

An Aroclor-containing test paint was prepared using a 1960s-era formula for preparing Aroclor 1254-amended chlorinated rubber marine paint formula (Monsanto ca. 1960). Subsequently, paint chips from this test paint were used in experiments to evaluate the rate and extent of leaching of PCBs into a freshwater system.

An estimate for the time at which the leaching rate of PCBs from paint chips ceased was determined from the experimental data. The total amount of PCBs that would partition from paint chips into water before cessation of leaching was also determined.

## Materials and Methods

### Aroclor 1254-Containing Test Paint

Aroclor-containing marine paints, including chlorinated rubber paints, are no longer commercially available. Therefore, an Aroclor 1254-containing chlorinated rubber‐based white marine paint was formulated for this study based upon a 1960s-era Monsanto Technical Bulletin (Monsanto ca. 1960) that specifies the components for the coating.

The specialty paint formulation company Walter Wurdack Inc. (St. Louis, MO) prepared the base paint for this study by formulating an Aroclor-free paint following as close as practical Monsanto specifications (Parlon™ chlorinated rubber (20 centipoise) [21.3 w%], Allnex Setal® 11–3466 long‐oil, oxidizing, alkyd resin [6.4 w%], R-900 titanium dioxide [26.7 w%], xylene [24.2 w%], Hi Sol 10 solvent [21.3 w%]). Pure Aroclor 1254 was purchased from Agilent Technologies (North Kingston, RI) and was added to the base paint at a concentration of ~ 6 weight percent Aroclor 1254 (liquid basis). This concentration is equivalent to ~ 12% Aroclor 1254 on a dry weight basis. The resulting mixture was determined to be 51.99% solids by weight.

The Aroclor 1254 concentration and the Aroclor 1254 compositional pattern of the test paint mixture were verified by replicate analyses. Five replicate subsamples of paint homogenate were extracted and analyzed by gas chromatography with electron capture detection (GC/ECD). Approximately 0.3 g of the test paint was diluted in dichloromethane (DCM). The extract was fortified with the surrogate compounds 2,4,5,6-tetrachloro-m-ylene (TMX) and decachlorobiphenyl (DCB), solvent exchanged into hexane, and acid cleaned using concentrated sulfuric acid following EPA Method 3665, *sulfuric acid/permanganate cleanup*. A portion of the extract was removed and fortified with the internal standard 1-bromo-2-nitrobenzene and analyzed by dual-column GC/ECD for determination of total Aroclor concentration following EPA Method 8082, *polychlorinated biphenyls by gas chromatography*. The instrument was calibrated using a seven-point Aroclor 1016/1260 calibration mix. Aroclor concentrations were determined using internal standard techniques. Sample concentrations were not corrected for surrogate recovery. Surrogate recoveries ranged from 85 to 99% with an average of 93%. Method Blanks accompanied each batch of 20 or fewer samples. Sample analysis data, sample-specific surrogate recovery data, and Method Blank results for these analyses are compiled in Supporting Information to this manuscript.

The concentrations of Aroclor 1254 measured in the test paint by GC/ECD compare favorably with the gravimetric mass of Aroclor 1254 added to the base paint (5.91% versus 6.18%, respectively). The relative standard deviation (RSD) for the five replicate measurements of the test paint was 4%. The qualitative gas chromatography patterns for the PCBs measured in the samples aligned with that of Aroclor 1254. These analytical data demonstrated that the Aroclor 1254 concentration and PCB congener distribution in the test paint were homogeneous.

The PCB congener chemistry of Aroclor 1254 is well understood (Frame et al. 1996). Aroclor 1254 contains a chlorinated biphenyl mixture that is 54% chlorine by weight. Rushneck et al. documented that 22 congeners (or congener pairs, in the case of co-eluting compounds) occur in Aroclor 1254 at concentrations greater than 1% of the mixture mass. As shown in Table 1, those 22 congeners make up 88.3% of the mass of Aroclor 1254 (Rushneck et al. 2004).

**Table 1:** PCB congener composition of Aroclor 1254

Congener	Number^1^	% Composition	Congener	Number^1^	% Composition
2,2',3,4',5–PeCB + 2,2',4,5,5'–PeCB	90 + 101	10.14	2,3,3',4,4'–PeCB	105	2.93
2,3,3',4',6–PeCB	110	9.10	2,2',3',4,5–PeCB	97	2.53
2,3',4,4',5–PeCB	118	6.84	2,2',3,3',4,6'–HxCB	132	2.47
2,2',3,5',6–PeCB	95	6.67	2,2',3,5'–TeCB	44	2.44
2,2',3,4,4',5'–HxCB + 2,3,3',4',5,6–HxCB	138 + 163	5.68	2,2',3,3',6–PeCB	84	2.43
2,2',5,5'–TeCB	52	5.36	2,2',3,5,5'–PeCB	92	1.41
2,2',3,4',5',6–HxCB	149	3.85	2,2',3,3',4,4'–HxCB	128	1.32
2,2',4,4',5,5'–HxCB	153	3.81	2,2',4,5'–TeCB	49	1.20
2,3',4',5–TeCB + 2',3,4,5–TeCB	70 + 76	3.67	2,2',3,4,4'–PeCB + 2,3,4,5,6–PeCB	85 + 116	1.20
2,2',4,4',5–PeCB	99	3.61	2,2',3,4,6–PeCB + 2,2',3,4',6–PeCB	88 + 91	1.01
2,2',3,4,5'–PeCB + 2',3,4,5,6'–PeCB	87 + 125	3.60	2,2',3,4,5,5'–HxCB	141	1.00

Table taken fromRushneck et al. (2004)

^1^PCB number per: Ballschmiter, K., Bacher, R., Fischer, M.R., Riehle, U., and Swerev, M. (1992). The determination of chlorinated biphenyls, chlorinated dibenzodioxins and chlorinated dibenzofurans by GC–MS. J. High Res. Chrom. 15: 260–270

**Fig. 1:**
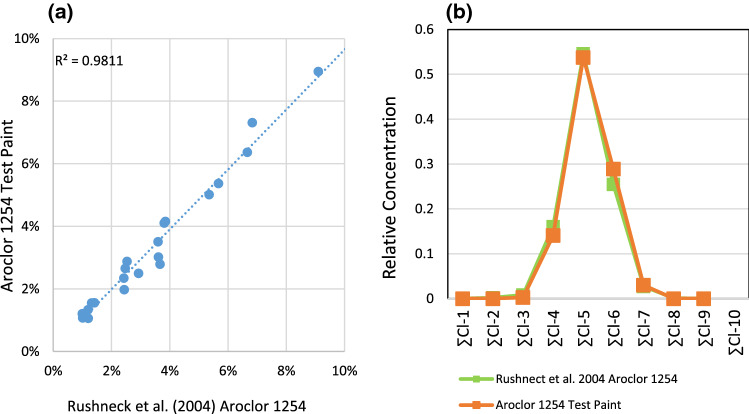
(**a**) Cross-plot of PCB congener composition for a reference Aroclor 1254 (Rushneck et al. 2004) vs that measured in the test paint prepared for this study and (**b**) compositional comparison of the same samples on a level of chlorination basis

The test paint was analyzed for 209 PCB congeners following EPA Method 680 (described below). Figure 1a plots the PCB congener composition for Rushneck et al.’s reported Aroclor 1254 composition (x-axis) versus the measured concentration of the same congeners in the test paint (y-axis). Only data for congeners measured at greater than 1% of the total PCB concentration are depicted. The test paint shows a strong compositional correlation to that reported by Rushneck et al. for Aroclor 1254 (*r*^2^ = 0.9811).

Similarly, Fig. 1b shows that, when expressed on a level of chlorination basis, the PCB composition between that reported for Aroclor 1254 by Rushneck et al. and that measured in the test paint in this study aligns well and is highly correlated (*r*^2^ = 0.9941).

### Dynamic Aging (Weathering) of Aroclor 1254-Containing Test Paint

Aroclor 1254-containing test paint was applied to carbon steel plates, dried for seven days, and dynamically aged over a 30-day period to simulate the natural weathering that paint applied to vessel hulls undergo during passage at sea. A dynamic aging process, patterned after Kojima et al. (2016), was used in this study to condition the paint-coated steel panels prior to generation of paint chips.

The test paint was applied to the front right and back left sides of a 30.5 cm × 35.6 cm steel panel, using a digital paint applicator (Gardner Digital Microm II) at a thickness of 8 mil, and allowed to air-dry for 7 days. Once air-dried, the panels were constantly rotated at 1.25 m/sec (50 rpm) in a 76-L polyethylene tank containing 68 L of Instant Ocean® artificial seawater, adjusted to salinity of 34 ppt. A zinc anode was added to top of each plate to minimize oxidation. This aging process was equivalent to a hull exposed to 3,200 km of ocean travel. The seawater was changed once per week over the 30-day aging period.

Only a small amount of the total PCBs in the aged paint chips leached from the panels during seawater aging. Direct measure of the total PCBs in the water used for panel aging indicated that approximately 0.1% of the total PCBs from the paint was found in the aqueous phase. Some of the measured PCBs were due to micro-particles of paint that sloughed off the panels during aging. This was confirmed by observation of a full-range Aroclor 1254 PCB congener pattern in unfiltered samples of the water, as compared to that for filtered water samples which contained a PCB congener pattern dominated by lower molecular weight, water-soluble compounds. Using the experimentally determined initial PCB leaching rate equation (Eq. 1), it is estimated that only 0.03% of the total PCBs actually leached from the painted panels into the dissolved phase during the dynamic aging process.

After the 30-day aging period, the painted panels were rinsed with deionized water (DI) and allowed to air-dry. Once dried, the aged paint was removed using a razor blade. The paint chips were mechanically reduced to a size that would pass through a 9.5-mm standard sieve.

The Aroclor concentration of the aged test paint chips was verified by replicate analyses of the material. Three subsamples of paint chips were extracted and analyzed by GC/ECD to verify the concentration and homogeneity of Aroclor 1254.

Approximately 0.1 g of paint chips was analyzed for total Aroclors following EPA Method 8082 as described above. The concentrations measured in the paint chips by GC/ECD compare favorably with the gravimetric mass of Aroclor 1254 added to the base paint (10.41% [surrogate corrected] versus 11.88%, respectively, corrected for percent solids). The precision in the three replicate measurements (0.25% RSD) demonstrates that the Aroclor 1254 concentration in aged paint chips was homogeneous and surrogate recoveries ranged from 81 to 98% with an average of 90%.

### Test Paint Chip Leaching Experiment

A time-series leaching experiment was conducted to evaluate the rate of PCB release and extent of mass release of PCBs from the test paint chips under simulated dynamic, shallow water conditions. The experiment was patterned after work by the US Navy, which studied the leaching of PCBs from various solid materials used in the fabrication of US Navy vessels (George et al. 2005).

Approximately 0.1 g of the ~ 12% (dry weight) Aroclor 1254-containing paint chips was placed in a stainless steel 74-µm mesh cage (Utah Biodiesel Company) and submerged in 900 mL of deionized water in a 1-L pre-cleaned amber glass leaching vessel containing a Teflon™-coated magnetic stir bar. The leaching vessel was placed in a water bath maintained at 25 °C. To simulate dynamic flow, each vessel was placed on a magnetic stirrer set at 80 rpm which produced a slight dimple but no measurable vortex. After initiation of the experiment, leachate was harvested on Days 3, 7, 14, 21, 28, 35, 42, 49, 56, 63, 90, 120, 150, and 180.

At the time of collection, the cage containing the paint chips was removed and placed into a new 1-L leaching vessel containing fresh deionized water and returned to the water bath for continued leaching.

After each collection of leachate, the water was filtered into an Erlenmeyer flask through a Buchner funnel fitted with a 0.45-µm Teflon™ filter under gentle vacuum. The leaching vessel was rinsed three times with deionized water, and the rinsate was passed through the filter to remove any fine particulates that may have passed through the cage to ensure a quantitative transfer from the vessel to the filter. The water was transferred to a separatory funnel, fortified with ^13^C-labled PCB congener surrogates (^13^C-PCB 19 and ^13^C-PCB 202), and serially extracted three times with DCM following EPA Method 3510, *separatory funnel liquid–liquid extraction*. The Erlenmeyer flask and sample vessel were rinsed with the first addition of solvent. The combined extract was concentrated, solvent exchanged into hexane, and cleaned with sulfuric acid following EPA Method 3665, *sulfuric acid/permanganate cleanup*. All extracts were fortified with ^13^C-labled PCB congener internal standards (^13^C-PCB 15 and ^13^C-PCB 180) for purposes of determining extraction efficiency. Sample extracts were analyzed for 209 PCB congeners using EPA Method 680 *determination of pesticides and PCBs in water and soil/sediment by gas chromatography/mass spectrometry* with the mass spectrometer operated in the selected ion monitoring mode. Samples were analyzed using an Agilent HP6890 GC equipped with a Restek RTX-PCB 60-m × 0.18 mm ID, 0.18 µm film thickness, fused-silica capillary column. The concentration of the 209 PCB congeners was quantified versus internal standards, and the congener concentrations were quantified using average response factors generated from the minimum of a six-point multi-level calibration curve for the 209 PCB congeners. Sample concentrations were not corrected for surrogate recovery. Method Blanks accompanied each batch of 20 or fewer samples. Sample results, sample-specific surrogate recovery data and Method Blank results for these analyses are compiled in Supporting Information to this manuscript. Surrogate recoveries ranged from 62 to 98% with an average of 82%.

## Results and Discussion

Total PCBs—defined as the sum of the measured 209 congeners—were measured periodically in triplicate samples of water in contact with the test paint chips over a 6-month period. There were a total of 14 sampling events. The first sampling event was taken 3 days after initiation of the leaching experiment. Beginning at Day 7, leachate samples were taken at 7-day intervals until Day 63. Subsequently, four additional samples were taken at 30-day intervals, terminating at Day 180 (see Supplemental Information Section). A summary of the total PCB concentration data collected over the course of the experiment is presented in Table 2.

**Table 2:** Total PCBs^1,2^ in test paint chip leachate measured over time

Time	Rep1	Rep 2	Rep 3	Average	Std Dev^3^	%RSD^4^
(Days)	ng-L^−1^-g^−1^	ng-L^−1^-g^−1^	ng-L^−1^-g^−1^	ng-L^−1^-g^−1^	ng-L^−1^-g^−1^	-
3	55,424	66,342	42,694	54,820	11,835	22
7	49,638	46,464	39,154	45,085	5,376	12
14	27,440	30,095	27,032	28,189	1,663	6
21	16,762	25,369	24,669	22,267	4,780	21
28	16,851	19,949	20,875	19,225	2,107	11
35	17,690	20,745	19,661	19,365	1,549	8
42	13,296	15,970	15,514	14,926	1,431	10
49	11,276	19,199	16,403	15,626	4,018	26
56	10,255	15,318	17,231	14,268	3,605	25
63	10,288	13,748	13,946	12,660	2,057	16
90	17,437	20,253	23,181	20,290	2,872	14
120	19,000	22,479	19,036	20,172	1,998	10
150	16,859	14,734	19,560	17,051	2,419	14
180	12,626	18,074	19,339	16,680	3,567	21

^1^Total PCBs: sum of the measured 209 congeners

^2^Units expressed as nanograms per liter of total PCBs per gram of paint chips

^3^Std Dev: standard deviation

^4^%RSD: percent relative standard deviation

Triplicate analyses were performed at every sampling interval. The precision in the triplicate measurements, expressed as percent RSD, ranged from 6 to 26% and averaged 15%RSD*.*

### General Dissolution Behavior

**Fig. 2:**
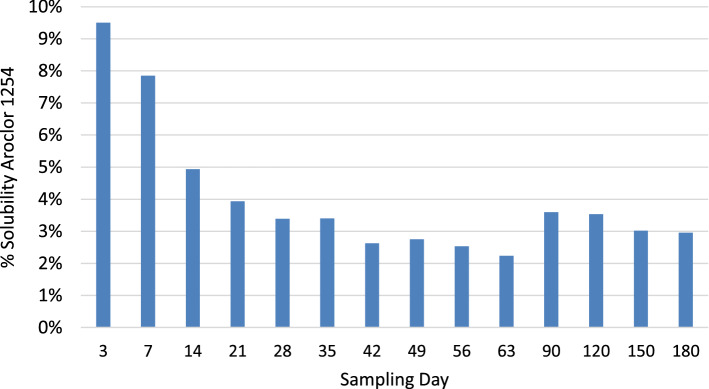
Measured solubility of Aroclor 1254 in test paint chips as a percentage of Aroclor 1254 solubility in pure water over the course of the leaching experiment

The water solubility of Aroclor 1254 is approximately 70 µg/L (Lee et al. 1979). The concentration of total PCBs measured at any time interval during the leaching experiment was found to be notably lower than the reported solubility of Aroclor 1254 and decreased over the course of the experiment (Fig. 2). This is consistent with reports that pure Aroclor formulations require on the order of five months to reach equilibrium with water (Lee et al. 1979). The highest relative solubility of Aroclor 1254 occurred during the earliest phases of the leaching experiment and then decreased systematically with time until Day 63. There was a modest increase in the relative solubility for Aroclor 1254 for Days 90, 120, 150, and 180. This is a reflection of the move to 30-day sampling intervals after Day 63, resulting in more time between sampling intervals after Day 63 to allow more of the Aroclor 1254 to partition into the water phase.

In heterogeneous systems (as is the case for paint chips in contact with water), non-equilibrium conditions can result from hindered solute transport caused by kinetically limited adsorption/desorption pathways (e.g., structural constraints in heterogeneous systems that influence the rate of solute transport [Jardine 2005]).

The implication of this observation is that the apparent leaching rates tracked over the course of the 180-day experiment are not equilibrium (saturation) limited, i.e., the rate of leaching measured over the course of the experiment is a reflection of the true dynamic leaching of PCBs from the paint chips. However, in the latter parts of the experiment (> 50 days), the rates of dissolution were very slow and approximated a pseudo-equilibrium condition (Scheckel and Impellitteri 2004).

### Rate and Extent of PCB Leaching

**Fig. 3:**
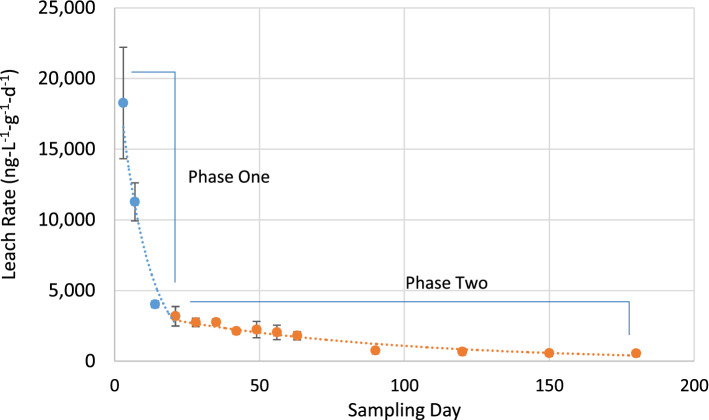
Rate of leaching of PCBs from Aroclor 1254-containing test paint chips. The data could be divided into two phases. Phase One: between Day 0 and Day 21 exhibiting the largest apparent dissolution rates, and Phase Two: between Day 21 and Day 180, representing a substantially slower rate of release of total PCBs over time. Data shown are average ± 1 standard deviation in triplicate measurements

The rate of leaching of PCBs from the paint chips over the course of the experiment decreased rapidly with time (Fig. 3). The leaching rate of PCBs from the paint chips between the initiation of the experiment and Day 3 was 18,273 ng-L^−1^-g^−1^-d^−1^. By the end of the experiment, the leaching rate between Day 150 and Day 180 had fallen to 556 ng-L^−1^-g^−1^-d^−1^, a decrease of more than a factor of 30 from the initiation of the experiment.

No one simple mathematical formula was found to adequately model the dissolution rate data over the full range of the experiment. Empirically, the data could be divided into two phases—Phase One, between Day 0 and Day 21, exhibited the largest apparent dissolution rates. Phase Two, between Day 21 and Day 180, represented a substantially slower rate of release of PCBs over time (Fig. 3). Both phases could be fit with simple exponential equations with a high degree of reliability.

The best-fit exponential equation for Phase One was:

1 $$y = 22,442e^{{ - 0.101x}} \left( {r^{2} = 0.9373} \right).$$

The best-fit exponential equation for Phase Two was:

2 $$y = 3,766e^{{ - 0.012x}} \left( {r^{2} = 0.9061} \right).$$

Since Eq. 2 models the terminal phase of PCBs leaching from the paint chips, application of that equation provides an estimate for the time at which the leaching rate of PCBs from the paint chips would be equal to zero. Using Eq. 2 and substituting zero for the leaching rate, it is estimated that the time at which the leaching rate of PCBs from the paint chips would cease is 1,150 days (range: 1,060 days to 1,145 days among triplicate experiments). Thus, barring no disruption of the physical characteristics of the paint chips (e.g., surface abrading, fracturing, etc.), PCBs would cease leaching from the paint chips after approximately 3.1-year exposure to water.

The percentage of PCBs predicted to remain in the paint matrix after the cessation of leaching was computed by (a) calculating the mass of PCBs that leached from the experimental system from direct measurement data between Day 0 and Day 180 and combining that with (b) the mass of PCBs predicted to leach from the PCB-paint chips between Day 180 and the estimated time of cessation of leaching (Day 1,150). Based on that data, it is estimated that more than 99% of the PCBs in the PCB-paint chips remain trapped in the paint matrix at the cessation of leaching.

### PCB Congener Distribution in the Dissolved Phase

The dissolved-phase PCB congener composition was tracked over the course of the 180-day paint chip leaching experiment in order to determine how the dissolved PCB congener distribution compared to the native Aroclor 1254 contained in the paint chips, and to ascertain if there was a change in the distribution of dissolved-phase PCB congeners over time.

**Fig. 4:**
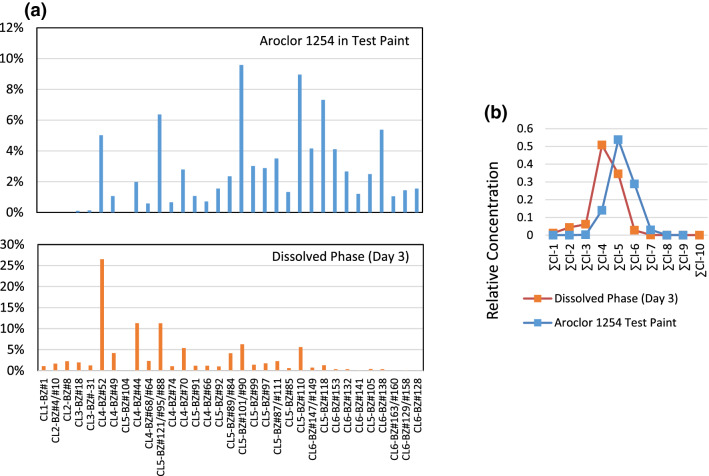
(**a**) Congener distribution for Aroclor 1254 measured in test paint (top) compared to congener distribution in dissolved phase in contact with the test paint, and (**b**) compositional comparison of the same samples on a level of chlorination basis

From the outset of the experiment until the last sampling at Day 180, the dissolved-phase PCB congener assemblage was different than that of the native Aroclor 1254 contained in the paint chips. Figure 4a contrasts the PCB congener distribution for Aroclor 1254 measured in the Aroclor 1254-containing paint chips (top), to that of the congener distribution that had leached from the paint chips into the dissolved phase at Day 3 of the experiment. (Note that only those congeners that are present at concentrations greater than 1% of the total PCBs in either the Aroclor 1254 or the dissolved phase are shown.)

It is evident that there is a marked difference between the dissolved phase and native Aroclor 1254 congener distributions, where the aqueous-phase PCB assemblage is dominated by congeners of lower levels of chlorination compared to the native Aroclor 1254. The congener composition of native Aroclor 1254 has an average level of chlorination of 5.0; in fact, the congeners containing 5 chlorines per molecule (Cl-5 congeners) compose more than 63% of the mass of Aroclor 1254. In the dissolved phase, the PCB composition shifts to a mixture of lower molecular weight congeners and found to be composed of approximately 53% Cl-4 congeners, and with an average level of chlorination of 4.1.

The overall change in PCB congener composition of the dissolved phase compared with that of Aroclor 1254 in the test paint chips is evident when comparing both on a level of chlorination basis (Fig. 4b). In this illustration, congeners of the same level of chlorination are summed, and the total for each level of chlorination is expressed as a percentage of total PCB. Here, the marked shift in composition from Aroclor 1254 (where Cl-5 congeners dominate) to the dissolved phase (where Cl-4 congeners dominate) is evident.

**Fig. 5:**
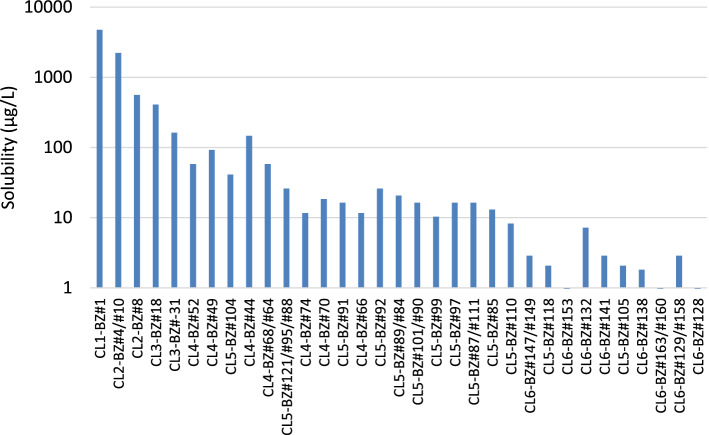
Estimated solubility of PCB congeners reported by Abramowitz and Yalkowsky (1990). Congeners shown occur at concentrations greater than 1% of total PCB in either Aroclor 1254 or its measured dissolved phase

The observation of a shift to a predominance of lower-chlorinated PCB congeners in the dissolved phase compared to a pure Aroclor formulation is well recognized (U.S. EPA 1980). The compositional shift observed in this experiment between the Aroclor 1254 in the test paint chips and the dissolved phase is a reflection of the greater water solubility of lower-chlorinated PCB congeners compared to higher-chlorinated analogs. Figure 5 depicts the solubilities for the PCB congeners found to comprise 1% of total PCB in either the PCB paint or the dissolved phase. The solubilities range from 4,739 µg/L (Cl1-BZ#1) to 0.9 µg/L (Cl6-BZ#128) (Abramowitz and Yalkowsky 1990).

The relative concentration of PCB congeners that partition from an Aroclor into the dissolved phase is a function of the mole fraction of the Aroclor 1254 congeners in the paint chips, and the congener’s individual solubility. PCB congener solubility data and Aroclor 1254 compositional data were used to estimate the congener distribution of the water-soluble fraction of Aroclor 1254 in the test paint using the Raoult’s law equation:

3 $$C_{w} = {\text{ }}X_{o} \bullet S~$$

whereC_w_ = effective solubility; X_o_ = mole fraction of the PCB congener; S = solubility of the pure PCB congener (Abramowitz and Yalkowsky 1990).

**Fig. 6:**
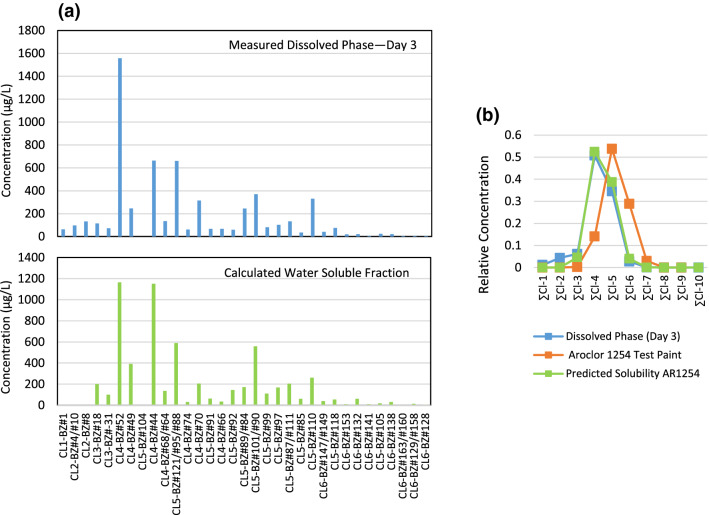
PCB congener distributions (**a**) measured in the leaching experiment dissolved phase compared to those predicted for the water-soluble fraction of Aroclor 1254 in the test paint and (**b**) PCB composition expressed as level of chlorination for the Aroclor 1254-containing test paint, and its measured and predicted water-soluble fractions

Figure 6a shows the comparison of the measured dissolved-phase PCB concentration at Day 3 of the leaching experiment with the predicted water-soluble fraction of Aroclor 1254 in the test paint. While there are some differences in the relative distributions of PCB congeners between actual and predicted, there is overall similarity between the patterns of PCB congeners. When PCB composition is expressed as level of chlorination (Fig. 6b), the similarity in the PCB congener assemblage for the measured dissolved-phase matches well with that of the predicted water-soluble phase of Aroclor 1254 (*r*^2^ = 0.9866).

The data demonstrate that the pattern of PCB congeners that leach from the Aroclor 125-containing test paint chips in this experiment is explained by solubility-driven partitioning of PCB congeners from the Aroclor 1254 in the paint chips and not by some other mechanism (e.g., sloughing of paint micro-particles from the test paint chips that escape into the aqueous phase, and which are subsequently measured as waterborne PCBs).

**Fig. 7:**
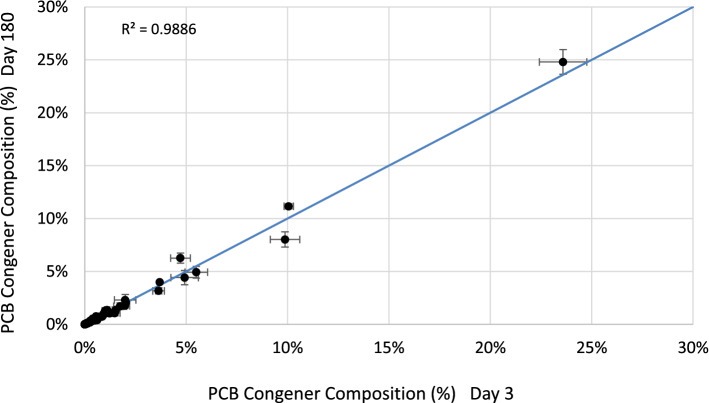
The concentration of leachate dissolved-phase PCB congeners at Day 3 vs Day 180 is highly correlated and demonstrates that the relative distribution of congeners in the dissolved phase remained constant over the course of the leaching experiment

While the rate of PCB leaching decreased rapidly over the course of the 180-day leaching experiment, the distribution of PCB congeners in the dissolved phase remained constant. Figure 7 shows a cross-plot of the dissolved-phase concentrations of PCB congeners (as percentage of total PCBs) measured at the beginning of the experiment (Day 3) and at the termination of the experiment (Day 180). The data fall along the 1:1 composition line and correlate highly (*r*^2^ = 0.9866). There was no evidence for differential rates of leaching among the PCB congeners from the paint matrix over the course of the experiment.

## Conclusions

The leaching of PCBs from Aroclor 1254-containing paint chips into pure water decreased rapidly and exponentially with time. A numerical best-fit to the leaching rate data indicated that PCBs would cease leaching from the paint chips after 1,150 days (range: 1,060 days to 1,145 days). At the cessation of leaching, more than 99% of the PCBs remains trapped in the paint chip matrix.

The PCBs that leached from Aroclor 1254-containing paint chips in this study resulted in a dissolved-phase congener assembly notably different than that found in the native Aroclor 1254 in the test paint. Over the course of leaching, the assemblage of PCB congeners in the dissolved phase remained constant over time. In the aqueous phase, the PCB congener assemblage was dominated by lower level of chlorination, lower molecular weight congeners compared to the native Aroclor 1254. In native Aroclor 1254, the Cl-5 congeners composed more than 50% of the mass of the Aroclor. In the dissolved phase, the PCB composition shifted to a mixture of relatively lower molecular weight congeners and was found to be composed of more than 50% Cl-4 compounds. This compositional shift is a reflection of the greater water solubility of the lower-chlorinated PCB congeners compared to higher-chlorinated analogs.

## Supplementary Information

Below is the link to the electronic supplementary material.

Supplementary file1 (XLSX 308 KB)
